# Optimal Dimensioning of Retaining Walls Using Explainable Ensemble Learning Algorithms

**DOI:** 10.3390/ma15144993

**Published:** 2022-07-18

**Authors:** Gebrail Bekdaş, Celal Cakiroglu, Sanghun Kim, Zong Woo Geem

**Affiliations:** 1 Department of Civil Engineering, Istanbul University-Cerrahpasa, Istanbul 34320, Turkey; 2 Department of Civil Engineering, Turkish-German University, Istanbul 34820, Turkey; cakiroglu@tau.edu.tr; 3 Department of Civil and Environmental Engineering, Temple University, Philadelphia, PA 19122, USA; sanghun.kim@temple.edu; 4 Department of Smart City & Energy, Gachon University, Seongnam 13120, Korea

**Keywords:** machine learning, optimization, structural design

## Abstract

This paper develops predictive models for optimal dimensions that minimize the construction cost associated with reinforced concrete retaining walls. Random Forest, Extreme Gradient Boosting (XGBoost), Categorical Gradient Boosting (CatBoost), and Light Gradient Boosting Machine (LightGBM) algorithms were applied to obtain the predictive models. Predictive models were trained using a comprehensive dataset, which was generated using the Harmony Search (HS) algorithm. Each data sample in this database consists of a unique combination of the soil density, friction angle, ultimate bearing pressure, surcharge, the unit cost of concrete, and six different dimensions that describe an optimal retaining wall geometry. The influence of these design features on the optimal dimensioning and their interdependence are explained and visualized using the SHapley Additive exPlanations (SHAP) algorithm. The prediction accuracy of the used ensemble learning methods is evaluated with different metrics of accuracy such as the coefficient of determination, root mean square error, and mean absolute error. Comparing predicted and actual optimal dimensions on a test set showed that an $R^{2}$ score of 0.99 could be achieved. In terms of computational speed, the LightGBM algorithm was found to be the fastest, with an average execution speed of 6.17 s for the training and testing of the model. On the other hand, the highest accuracy could be achieved by the CatBoost algorithm. The availability of open-source machine learning algorithms and high-quality datasets makes it possible for designers to supplement traditional design procedures with newly developed machine learning techniques. The novel methodology proposed in this paper aims at producing larger datasets, thereby increasing the applicability and accuracy of machine learning algorithms in relation to optimal dimensioning of structures.

## 1. Introduction

Retaining walls are a ubiquitous element in structural design. Due to their relatively large dimensions, optimizing their dimensions can lead to significant gains with construction costs. Furthermore, designing with minimum dimensions has certain advantages for ${\mathrm{CO}}_{2}$ emissions because of using the minimum amount of cement. Therefore, the application of advanced methodologies of optimization in the design of retaining walls has economic and environmental benefits.

Many newly developed optimization techniques have been used for structural optimization in recent years. Gomes [1] applied the particle swarm optimization technique to the mass optimization of steel trusses under frequency constraints. Similarly, Dede [2] analyzed the weight minimization of steel trusses using the teaching–learning-based optimization algorithm. Bekdaş et al. [3] used several metaheuristic optimization algorithms in the minimum total potential energy analysis of steel trusses. Bekdaş [4] applied the applications of harmony search, flower pollination, and teaching–learning-based optimization algorithms to minimizing the total construction cost associated with axially symmetric cylindrical reinforced concrete walls. Ocak et al. [5,6] optimized a tuned liquid damper device, which was used for lateral displacement control of structures using the adaptive harmony search algorithm. Ulusoy [7] applied the teaching–learning-based optimization algorithm to the problem of the fire-resistant design of timber-based roof structures. Cakiroglu et al. [8,9] showed that the social spider algorithm could affect the cost minimization problem for concrete-filled steel tubular columns. The optimum design of retaining walls has been investigated using various metaheuristic algorithms including the non-dominated sorting genetic algorithm (NSGA-II) [10], flower pollination algorithm [11], gravitational search algorithm [12], and harmony search algorithm [13,14,15].

In addition to various optimization algorithms, the application of machine learning techniques in structural design has been increasingly reported in the literature. Feng et al. [16] developed an XGBoost-SHAP machine learning model which estimates the shear strength of reinforced concrete shear walls. In their study, the database, which consisted of 434 samples, was split into a training and a test set in a 70% to 30% ratio. This split ratio is adopted by the majority of the studies in this field and it is based on the optimum split ratio established by Mangalathu et al. [17]. Somala et al. [18] showed that in the fundamental period estimation of masonry infilled reinforced concrete frames, Ensemble Learning Techniques such as Random Forest and XGBoost could outperform the existing empirical predictive models available in the literature. Ahmed et al. [19] developed a novel long short-term memory network with overlapping data for the accurate prediction of earthquake-induced damage in ductile and non-ductile frame structures. Ni et al. [20] generated fragility curves for buried pipelines using Lasso Regression Analysis. Bekdaş et al. [21] demonstrated the high accuracy of different Ensemble Learning Algorithms in predicting the optimal wall thickness of reinforced concrete cylindrical walls. Cakiroglu et al. [22] developed predictive models using Ensemble Learning Algorithms to estimate the axial load-carrying capacity of FRP-reinforced concrete columns.

The current paper deals with optimizing six key dimensions which define the dimensioning of a retaining wall. These dimensions are the length of the heel (X1), length of the toe (X2), the thickness of the stem at the top of the wall (X3), the thickness of the stem at the bottom of the wall (X4), the thickness of the foundation of the wall (X5), and the stem height of the wall (H). For each of them, a separate predictive model has been developed using four different Ensemble Learning Algorithms. Ensemble Learning Techniques have been demonstrated to have superior performance in terms of prediction accuracy in recent years in comparison to traditional methods of structural performance prediction. The dataset needed to train the predictive models has been created using the Harmony Search Algorithm. More than seventy thousand data samples have been created, where each one of these data points corresponds to an optimum design configuration. Every data sample in this dataset contains, in addition to the six geometric variables which define the retaining wall geometry, the soil density (γ), surcharge loading (q), soil friction angle (ϕ), the unit cost of concrete (C_c_), and the soil bearing capacity (q_z_).

Optimal dimensioning of retaining walls can lead to significant gains in terms of cost and environmental protection. In recent years, various optimization techniques have been demonstrated for minimizing the construction cost associated with retaining walls. On the other hand, machine learning algorithms are increasingly being used in the prediction of structural performance. However, it is necessary to train these predictive algorithms using large datasets for their accuracy. The availability of large-enough datasets has been a major bottleneck in the development of accurate predictive machine learning models for structural design in the recent years. Most of the research in this area has been conducted using datasets in the order of magnitude of a thousand data samples or fewer. To overcome this limitation, the current paper demonstrates a novel technique to generate significantly larger datasets with the help of optimization algorithms. The current paper is unique in its combination of metaheuristic optimization with machine learning models to obtain predictive models that can determine optimal dimensions of a retaining wall under various loading and soil conditions. The novelty of the paper is the usage of a well-established optimization methodology for the generation of large datasets that can be used in the training of machine learning models.

## 2. Methods of Optimization and Predictive Model Development

**Figure 1:**
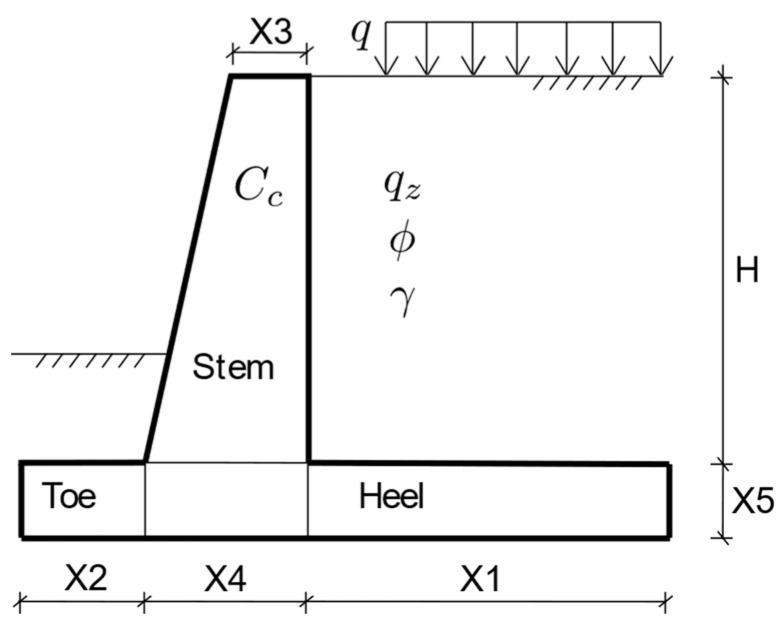
Retaining wall dimensions.

The current paper demonstrates the application of the harmony search algorithm in generating large datasets consisting of optimum design configurations. These design configurations consist of six key geometric variables which define the geometry of a retaining wall in addition to soil properties, concrete unit cost, and applied surcharge load. The variables of retaining wall geometry are shown in Figure 1. After generating a large dataset with more than seventy thousand combinations of design variables, four different machine learning models are trained based on this dataset. The following sections describe optimization and machine learning techniques.

### 2.1. Harmony Search Algorithm

The application of metaheuristic optimization algorithms to structural optimization is an active area of research. Among a large number of metaheuristic algorithms, the harmony search (HS) algorithm is one of the most widely used and established techniques, and applied to numerous areas such as structural design [23], water network design [24], flood model calibration [25], economic load dispatch [26], concrete mix proportion design [27], chaotic systems [28], timetabling [29], weapon target assignment [30], stock price prediction [31], mobile network security [32], COVID-19 detection from CT scans [33], and subway ventilation [34].

The technique is based on the incremental improvement of an initial population of randomly generated solution candidates, also called the harmony memory matrix. In the case of cost optimization of the retaining wall, the solution candidates are vectors consisting of variables such as the wall geometry, soil properties, unit cost of material used in the retaining wall construction, and the external loads, as shown in Equation (1) where harmony memory size (HMS) denotes the size of the population of candidate solution vectors.

(1) $$HM=\left[\begin{array}{cccccccccccc} X1^{1} & X2^{1} & X3^{1} & X4^{1} & X5^{1} & H^{1} & q_{z}^{1} & q^{1} & \gamma^{1} & \phi^{1} & C_{c}^{1} & f\left(x^{1}\right)\\ X1^{2} & X2^{2} & X3^{2} & X4^{2} & X5^{2} & H^{2} & q_{z}^{2} & q^{2} & \gamma^{2} & \phi^{2} & C_{c}^{2} & f\left(x^{2}\right)\\ \vdots & \vdots & \vdots & \vdots & \vdots & \vdots & \vdots & \vdots & \vdots & \vdots & \vdots & \vdots \\ X1^{HMS} & X2^{HMS} & X3^{HMS} & X4^{HMS} & X5^{HMS} & H^{HMS} & q_{z}^{HMS} & q^{HMS} & \gamma^{HMS} & \phi^{HMS} & C_{c}^{HMS} & f\left(x^{HMS}\right) \end{array}\right]$$

In Equation (1), each row of the harmony memory matrix (HM) contains the components of a candidate solution vector. The last column of the HM contains the output of a function f that takes a candidate solution vector as its argument and returns the performance of the solution vector. In the case of cost optimization, the output of f is the total cost of material used in constructing the retaining wall. Based on their performances, the solution vectors are ranked, and the best- and worst-performing members of the population are determined. In each HS iteration, the solution vectors are updated according to the steps shown in Equations (2)–(5).

(2) k=int(rand·HMS), rand∈(0,1)

(3) $$x_{i,\mathrm{new}}=x_{i,\min}+\mathrm{rand}\cdot \left(x_{i,\max}-x_{i,\min}\right),\;\mathrm{if}\;\mathrm{HMCR}\;>\;\mathrm{rand}$$

(4) $$x_{i,\mathrm{new}}=x_{i,k}+\mathrm{rand}\cdot \mathrm{PAR}\cdot \left(x_{i,\max}-x_{i,\min}\right),\;\mathrm{if}\;\mathrm{HMCR}\;\leq \;\mathrm{rand}$$

(5) $$\mathrm{HMCR}=0.5\left(1-\frac{i}{\max\left(i\right)}\right),\mathrm{PAR}=0.05\left(1-\frac{i}{\max\left(i\right)}\right)$$

HMCR and PAR in Equations (2)–(5) are the harmony memory consideration rate and the pitch adjustment rate, respectively. After each modification step, the newly generated solution vectors are ranked against the existing vectors. Among these vectors, the ones that perform better than the vectors of the previous iteration replace those worse-performing vectors. In the process of generating new solution candidate vectors, the constraints of optimization are regarded based on design codes for retaining walls so that the new design has enough capacity to resist the applied loads. The details of the HS algorithm and its different variants can be found in [35].

### 2.2. Machine Learning Methodologies

**Figure 2:**
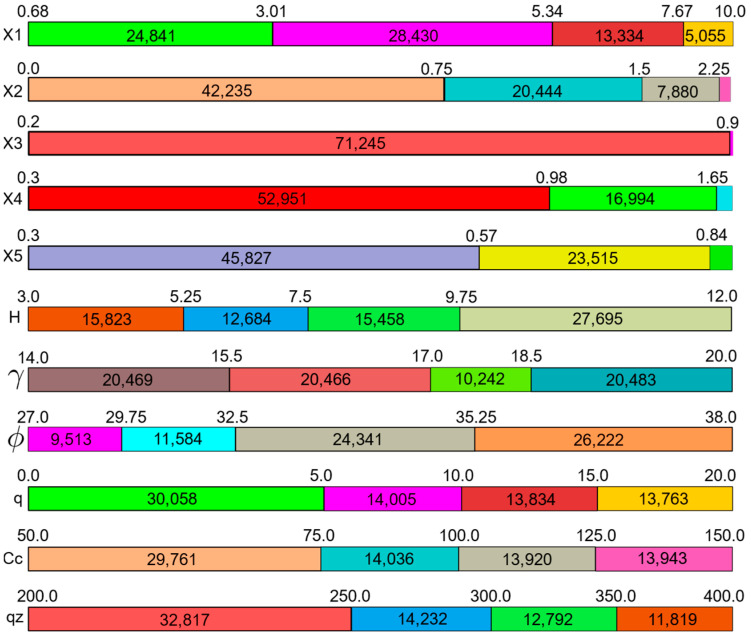
Design variable ranges in the dataset.

The database of optimum design combinations generated through the HS algorithm has been used in training predictive models. The design variables included in this dataset and their ranges are shown in Figure 2, where the values that each design variable takes are split into four different subgroups. For each one of these groups, the total number of samples belonging to that group is written inside the horizontal bars and the subgroup ranges are written above the subgroup boundaries. In Figure 2, the length of a subgroup indicates the percentage of the samples belonging to that group inside of the entire dataset.

Figure 2 shows the concrete unit price (C_c_) ranging between 50 and 150 USD/m^3^. It can be observed that the majority of cases were within the 75–150 USD/m^3^ range. The entire range of unit prices for concrete corresponds to a compressive strength of 16 to 50 MPa, which includes the compressive strengths of most commonly used concrete classes, excluding high strength concrete [36,37]. For the details of the correlation between the soil friction angles included in this study with the other soil properties and the soil classification, the reader is referred to [38].

The predictive models in this paper were generated using the XGBoost, Random Forest, LightGBM, and CatBoost algorithms. These models are further analyzed using the SHapley Additive exPlanations (SHAP) methodology. The following sections show a summary of the theoretical background of these methods.

#### 2.2.1. Extreme Gradient Boosting (XGBoost)

The XGBoost algorithm is a decision tree-based method that has the capability of scaling to large datasets with billions of samples. The decision tree technique starts with testing a root criterion and recursively branches into leaf nodes, testing further criteria, ultimately reaching a terminal node that contains the prediction. The algorithm controls overfitting by using a special regularization technique. The objective of the algorithm is to obtain mapping between the input vectors $x^{i}$ and the output values $y^{i}$ as shown in Equations (6) and (7), where L is the loss function, $f_{k}$ is a weak learner, $\alpha_{k}$ is the learning rate, T is the number of leaves, $w_{k}$ are the leaf weights, and γ and λ are the penalty coefficients [16,39].

(6) $${\hat{y}}^{i}=\phi \left(x^{i}\right)=\overset{K}{\underset{k=1}{\sum }}\alpha_{k}f_{k}\left(x^{i}\right)$$

(7) $$L\left(\phi \right)=\underset{i}{\sum }{\left(y^{i}-{\hat{y}}^{i}\right)}^{2}+\underset{k}{\sum }\gamma T+\frac{1}{2}\lambda \left|\left|w_{k}\right|\right|$$

#### 2.2.2. Random Forest

The Random Forest technique combines the predictions of an ensemble of single decision trees. The algorithm implements bagging and random feature selection techniques such that every decision tree in the ensemble is built using a bootstrap sample of the training set and the mean value of the individual tree predictions determines the overall predictive model prediction. In every node split, a random subset of features is selected for tree building. The random forest model can be summarized as in Equation (8), where ${\hat{m}}_{j}$ stands for a single decision tree [40,41,42].

(8) $$\hat{m}\left(x\right)=\frac{1}{M}\overset{N}{\underset{i=1}{\sum }}{\hat{m}}_{j}\left(x\right)$$

#### 2.2.3. Light Gradient Boosting Machine (LightGBM)

LightGBM is another decision tree-based algorithm, where the leaf-wise generation of the predictive model enables the creation of more complex trees. This is a version of the gradient boosting algorithm with improved computational speed and better accuracy. Using the Gradient-based One-Side Sampling (GOSS) method, LightGBM can handle large datasets. The Exclusive Feature Bundling (EFB) method makes it possible to handle datasets with a large number of design features in a more efficient way compared to the basic gradient boosting decision tree [43,44,45].

#### 2.2.4. Categorical Gradient Boosting (CatBoost)

CatBoost differentiates itself from the basic gradient boosting decision tree in that it is capable of dealing with categorical input features more efficiently. The built-in one-hot encoding capability of CatBoost can obtain target statistics from categorical features. Furthermore, the ordered boosting technique allows the CatBoost algorithm to overcome the gradient bias. Let $X_{i}=\left(x_{i,1},\ldots ,x_{i,m}\right)$ be an input vector consisting of m design features and $Y=\left(Y_{1},\ldots ,Y_{n}\right)$, the vector of labels. Let $\sigma =\left(\sigma_{1},\ldots ,\sigma_{n}\right)$ be a permutation. To reduce overfitting and use the entire dataset, the CatBoost algorithm uses a random permutation by substituting $x_{\sigma_{p},k}$ with the expression in Equation (9), where P is a prior value and a > 0 is its weight [46,47].

(9) $$\frac{\sum_{j=1}^{p-1}\left[x_{\sigma_{j},k}=x_{\sigma_{p},k}\right]Y_{\sigma_{j}}+a\cdot P}{\sum_{j=1}^{p-1}\left[x_{\sigma_{j},k}=x_{\sigma_{p},k}\right]+a}$$

#### 2.2.5. SHapley Additive exPlanations (SHAP)

The SHAP analysis is a great contribution to the explainability of the machine learning models, in that it enables a visual representation of the impact of each input variable on the predictive model outcome. Furthermore, Shapley values can measure the interdependencies between different input variables. The algorithm is based on game theory and uses the additive feature attribution, method where an output model is defined as a linear combination of simplified input vectors, as shown in Equation (10) [48].

(10) $$f\left(x\right)=g\left(x^{\prime }\right)=\phi_{0}+\overset{M}{\underset{i=1}{\sum }}\phi_{i}{x_{i}}^{\prime }$$

In Equation (10), the functions f and g are the original predictive model and the explanation model, respectively. M is the total number of input variables, **x** is a vector of input variables, $x_{i}$ are the simplified input vectors, and $\phi_{i}$ are the Shapley values. The Shapley values are calculated using Equation (11). Further details of the SHAP algorithm can be found in [49].

(11) $$\phi_{i}\left(f,x\right)=\underset{z^{\prime }\subseteq x^{\prime }}{\sum }\frac{\left|z^{\prime }\right|!\left(M-\left|z^{\prime }\right|-1\right)!}{M!}\left[f_{x}\left(z^{\prime }\right)-f_{x}\left(z^{\prime }\backslash i\right)\right]$$

## 3. Results

In this section, the predictions of four different Ensemble Learning Algorithms are compared to the actual optimum dimensions obtained through the harmony search method.

### 3.1. Comparison of the Model Predictions

**Figure 3:**
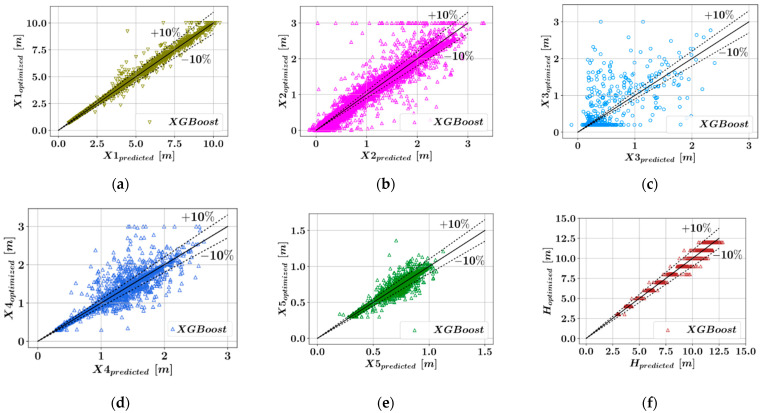
Comparison of the predicted and optimized dimensions using XGBoost. (**a**) X1, (**b**) X2, (**c**) X3, (**d**) X4, (**e**) X5, (**f**) H.

**Figure 4:**
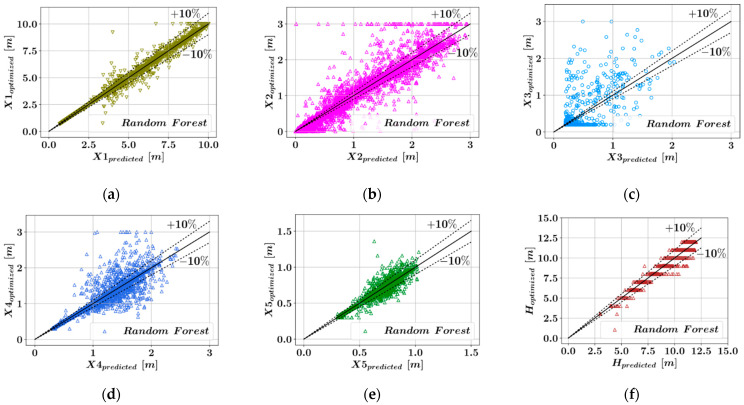
Comparison of the predicted and optimized dimensions using Random Forest. (**a**) X1, (**b**) X2, (**c**) X3, (**d**) X4, (**e**) X5, (**f**) H.

**Figure 5:**
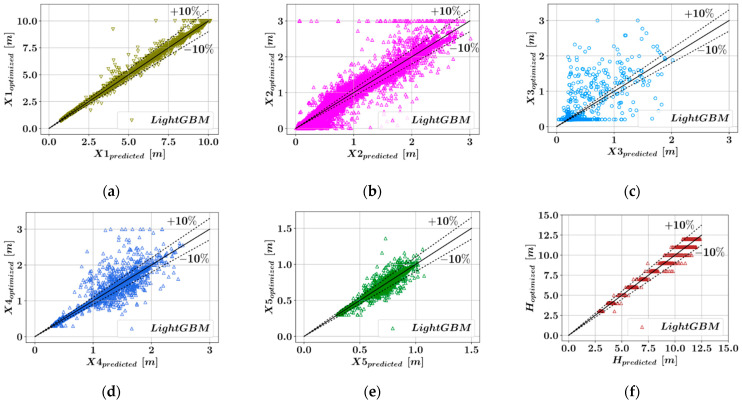
Comparison of the predicted and optimized dimensions using LightGBM. (**a**) X1, (**b**) X2, (**c**) X3, (**d**) X4, (**e**) X5, (**f**) H.

**Figure 6:**
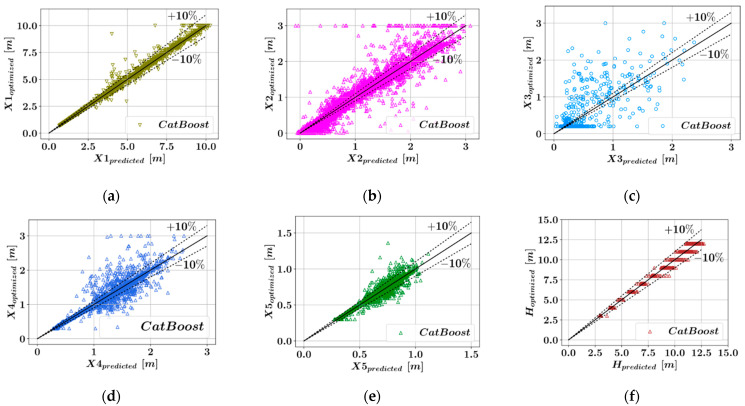
Comparison of the predicted and optimized dimensions using CatBoost. (**a**) X1, (**b**) X2, (**c**) X3, (**d**) X4, (**e**) X5, (**f**) H.

The comparisons have been visualized for all of the six key dimensions that describe the retaining wall geometry. For each algorithm and each dimension that is being predicted, the accuracy of the predictive models has been measured using three different metrics and listed in Table 1. In Figure 3, Figure 4, Figure 5 and Figure 6, the predicted optimum dimensions are plotted against the actual optimized dimensions. It can be observed that in the plots showing the predictions for X1, X5, and H, the points representing the different configurations are within a relatively narrow band, which indicates higher accuracy of prediction. In each one of these plots, the dotted ±10% lines can be seen, which indicates a 10% deviation from a perfect match between the predicted and actual optimal dimensions.

Table 1 shows that five out of the six parameters defining the wall geometry could be accurately predicted using the ensemble learning models. Among these models in Table 1, low R^2^ values were obtained for X3, since the database used in the training of the predictive models is mostly populated with samples where the X3 value is 0.2. This distribution of the design variable values was obtained after eliminating the design configurations for which the harmony search method did not converge to an optimum result within design constraints. Taking the average value of the metrics of accuracy corresponding to different dimensions, it can be seen from Table 2 that the CatBoost model has the best performance in terms of all three accuracy metrics. CatBoost was followed by Random Forest and LightGBM, whose performances were close to each other. Lastly, the XGBoost models had the lowest accuracy among the four predictive models.

**Figure 7:**
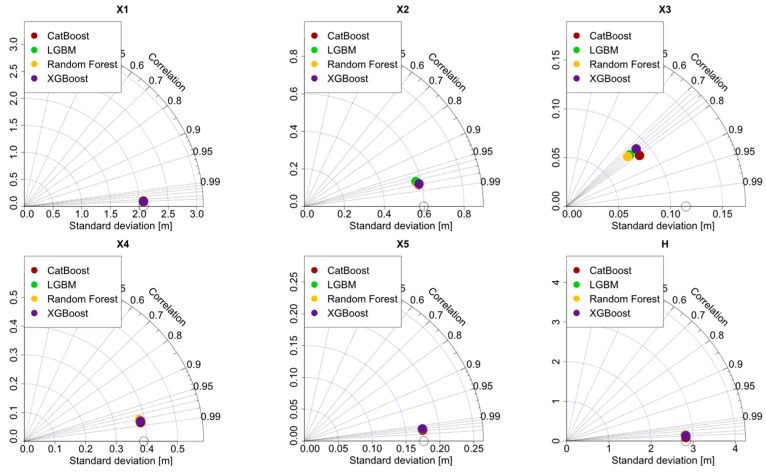
Taylor diagrams for the design variables.

The Taylor diagrams in Figure 7 show the model quality by using the Pearson correlation coefficient as the metric of accuracy. The equation for the calculation of the Pearson correlation is given in Appendix A. The prediction of each model is shown with a circle and the corresponding correlation coefficient is shown on the radial grid, which ranges from 0 to 1. Furthermore, for each predictive model as well as the original dataset, the corresponding standard deviation values are calculated and shown on both the horizontal and vertical axes. From Figure 7, it can be seen that for the design variables X1, X5, and H, the correlation coefficients were greater than 0.99 for all predictive models, which indicates excellent accuracy of prediction. For X2 and X4, the correlation values were 0.98 for all predictive models. The lowest correlation values were observed for X3 in the interval from 0.75 to 0.80, where the highest correlation values could be obtained through the CatBoost model. A summary of the predictive model performance can be observed in Table 2, where the average values of the error metrics are listed for all the models. According to Table 2, the CatBoost models have the highest accuracy on average.

### 3.2. SHAP Analysis

**Figure 8:**
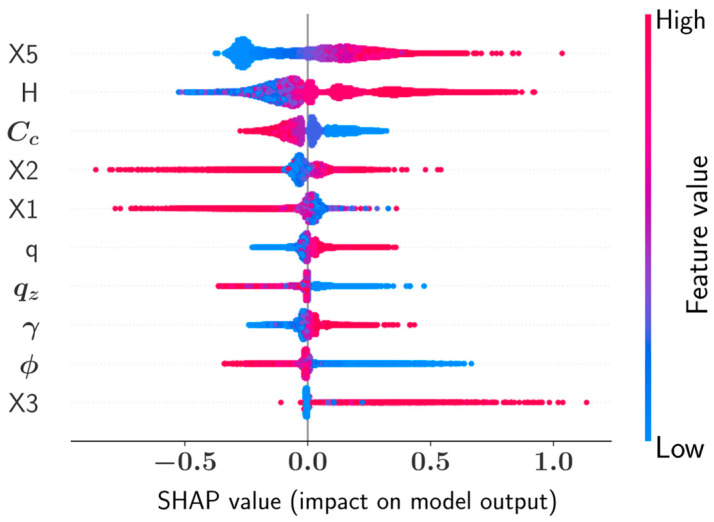
SHAP summary plot for X4.

The SHAP summary plots and feature dependence plots presented in this section provide an effective way of visualizing the impact of various design variables on the overall predictions of the machine learning models. The summary plot shown in Figure 8 is an information-rich representation of how ten different input variables affected the CatBoost model outcome for the prediction of the wall stem thickness at the bottom (X4). In Figure 8, each dot corresponds to a different sample in the database. The dot positions along the horizontal axis are related to the SHAP value of the variable such that greater positive values indicate an increasing effect on the model prediction and negative SHAP values indicate a decreasing effect on the model output. Furthermore, the magnitude of a variable in a sample is represented with color such that greater magnitudes are shown with the shades of blue and lower values are shown with the shades of red. According to Figure 8, the thickness of the wall foundation (X5) has the greatest impact on X4 such that increasing the X5 value also increases X4.

**Figure 9:**
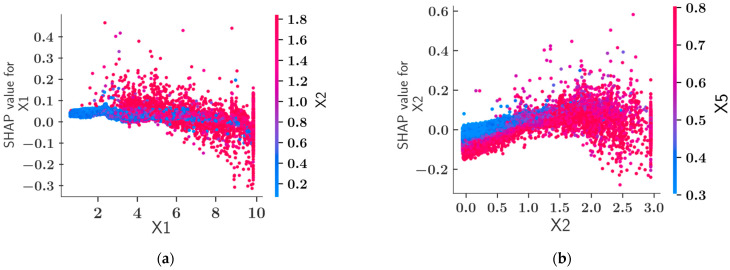

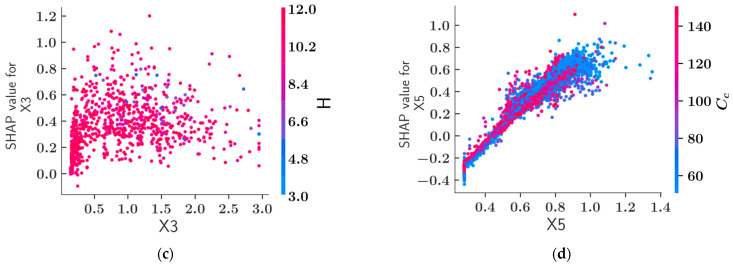
Feature dependence plots for the geometric variables. (**a**) CatBoost dependence plot for X1, (**b**) CatBoost dependence plot for X2, (**c**) CatBoost dependence plot for X3, (**d**) CatBoost dependence plot for X5.

The feature dependence plots in Figure 9 present further information about the interdependencies of the different input variables. Figure 9a shows that as the value of X1 increases, the SHAP value decreases. Therefore, its impact on the model output tends to decrease. Particularly when the length of the toe (X2) has a high value, this relationship between X1 and its impact is more pronounced. From Figure 9b, it can be observed that up to a certain value as X2 increases, its SHAP value increases regardless of the value of X5, which is the variable most dependent on X2. For X2 > 1.5, the impact of these variables decreases with its size when X5 has higher values shown with the shades of red. The relationship between the values of X5 and the impact of this variable on the model output can be observed in Figure 9d. For X5 < 1, the value of X5 and its impact are linearly proportional regardless of the value of $C_{c}$, which is the most dependent parameter on X5.

## 4. Discussion

The current paper presents a novel technique for the optimum dimensioning of retaining walls. A data-driven approach is presented using four different ensemble learning techniques. Predictive machine learning models have been generated using a large dataset obtained through optimization. The thickness of the retaining wall stem at the bottom has been used as the decisive parameter that determines the overall size and cost of the structure. The database necessary to develop the predictive models has been generated using the HS optimization technique. Using this technique, a large database with over seventy thousand data samples was generated where each data sample consists of an optimum combination of eleven design variables and the total construction cost associated with them. The prediction accuracy of the different models has been presented using root mean square error (RMSE), mean absolute error (MAE), coefficient of determination (R^2^), and Pearson correlation as the metrics of model performance. The highest prediction accuracy could be achieved by the CatBoost models followed by LightGBM, Random Forest, and XGBoost. The focus of this analysis was the optimization of the geometric dimensions of a retaining wall. The overall wall size and shape were described using six key geometric dimensions.

Previous studies in the area of optimal structural dimensioning mostly attempted to minimize structural cost or weight for a single load case [8,9]. More recent studies in the area attempted to develop general-purpose predictive models based on a dataset of structural configurations with known structural behavior [21,22]. However, the availability of experimental or numerical data describing the structural behavior is a major limiting factor in the training of robust predictive models since the size of the database used in the training of these predictive models is a decisive factor that effects to what extent these models could be used reliably. Furthermore, the range of design variables included in the dataset determines the accuracy of the predictions on new data samples. The current study differentiates itself from the previous ones by generating comprehensive predictive models that incorporate a large number of samples and design variables. Both the size of the dataset and the ranges of the design variables were selected so that these ranges would include most load cases with practical relevance. As a result, a dataset of 71,660 data samples was generated using the harmony search optimization algorithm, which is significantly larger than the datasets previously used in this field. The current paper demonstrates the possibility of generating significantly larger datasets using optimization techniques. This novel approach has the potential to overcome the data availability limitations associated with training machine learning models for structural dimensioning. Using this approach, the applicability of machine learning algorithms to the field of engineering design can be greatly enhanced.

From the SHAP summary plot, it could be observed that all geometric variables, except for the length of the heel (X1), have an increasing effect on the wall stem thickness at the bottom (X4). On the other hand, variables such as concrete unit cost, soil friction angle, and soil bearing capacity have a decreasing effect on X4 as their values increase. Furthermore, increasing the magnitude of the soil density (γ) was observed to have an increasing effect on X4. The thickness (X5) of the wall foundation was found to have the greatest impact on X4, whereas X3 was the variable with the least impact. The low impact of X3 on the model output can be attributed to the concentration of the X3 values around a single value in the entire database.

## 5. Conclusions

The availability of large datasets is crucial for the development of accurate predictive models in machine learning and particularly in structural dimensioning. The current paper shows the generation of a database consisting of 71,660 unique optimal combinations of six different geometric variables and five parameters that describe the material properties and external loads. The harmony search algorithm has been utilized to obtain these optimal configurations. The major outcomes of this paper can be summarized as follows:

- Among the four ensemble learning models developed in this paper, the highest overall prediction accuracy could be achieved by the CatBoost model, with a maximum coefficient of determination score of 0.999 for the prediction of the optimum stem height and an average $R^{2}$ score of 0.927, while the XGBoost models demonstrated, on average, the lowest prediction accuracy.
- In terms of computational speed, the LightGBM models demonstrated the best performance, with an average duration of 6.17 s for the training and testing, whereas the CatBoost models were an order of magnitude slower than the LightGBM models.
- The results of the SHAP analysis showed that the thickness of the retaining wall foundation (X5), the unit cost of concrete (C_c_), and the stem height of the wall have the greatest impact on the optimal design.
- The foundation thickness and concrete unit cost were found to be highly dependent on each other and a linear proportionality could be observed between the foundation thickness and the impact of this parameter on the optimal design configuration.

Further research in this area can be carried out by setting different material properties such as the compressive strength of concrete or the yield strength of steel as the optimization objective. Furthermore, the arrangement of the steel reinforcement can be included in future studies as a design variable or optimization objective. One of the limitations of the current study is that a certain range of unit prices is assumed during the generation of the dataset which represents the quality of concrete. However, fluctuations in concrete unit prices have not been considered. Furthermore, it should be noted that the developed ensemble learning models are only applicable within variable ranges included in the training dataset. For variable values outside these ranges, detailed structural analysis and optimization techniques should be applied on a case-by-case basis.

## Figures and Tables

**Table 1:** Prediction accuracy of the machine learning models.

Algorithm	Variable	R^2^	MAE	RMSE	Duration [s]
XGBoost	X1	0.977	0.0697	0.2988	16.49
	X2	0.958	0.0573	0.1319	19.12
	X3	0.562	0.0092	0.0759	16.89
	X4	0.967	0.0197	0.0708	17.88
	X5	0.988	0.0075	0.0192	17.62
	H	0.998	0.0907	0.1351	14.98
Random Forest	X1	0.997	0.0279	0.1091	65.02
	X2	0.958	0.0378	0.1220	62.47
	X3	0.559	0.0083	0.0762	94.74
	X4	0.960	0.0162	0.0776	66.19
	X5	0.989	0.0052	0.0188	61.61
	H	0.997	0.0702	0.1525	51.24
LightGBM	X1	0.998	0.0463	0.0989	5.86
	X2	0.947	0.0719	0.1383	5.68
	X3	0.566	0.0100	0.0756	6.27
	X4	0.966	0.0208	0.0725	5.59
	X5	0.989	0.0075	0.0186	7.36
	H	0.997	0.1051	0.1517	6.28
CatBoost	X1	0.998	0.0281	0.0860	85.31
	X2	0.960	0.0505	0.1189	73.05
	X3	0.642	0.0093	0.0687	74.85
	X4	0.971	0.0167	0.0660	85.76
	X5	0.991	0.0056	0.0170	90.40
	H	0.999	0.0524	0.0890	75.43

**Table 2:** Average predictive model accuracy and performance.

Algorithm	R^2^	MAE	RMSE	Duration [s]
XGBoost	0.9083	0.04235	0.12195	17.16
Random Forest	0.91	0.0276	0.0927	66.88
LightGBM	0.9105	0.0436	0.0926	6.17
CatBoost	0.92683	0.0271	0.07427	80.80

## Data Availability

The data presented in this study are available on request from the corresponding author.

## Appendix A

**Table A1:** Metrics of Model Accuracy.

Root mean square error (RMSE):	$\mathrm{RMSE}=\sqrt{\frac{\sum_{i=1}^{n}{\left(y_{i}-{\tilde{y}}_{i}\right)}^{2}}{n}}$
Coefficient of determination (R^2^):	
$R^{2}={\left(\frac{n\sum_{i=1}^{n}y_{i}{\tilde{y}}_{i}-\sum_{i=1}^{n}y_{i}\sum_{i=1}^{n}{\tilde{y}}_{i}}{\sqrt{n\sum_{i=1}^{n}y_{i}^{2}-{\left(\sum_{i=1}^{n}y_{i}\right)}^{2}}\sqrt{n\sum_{i=1}^{n}{\tilde{y}}_{i}^{2}-{\left({\tilde{y}}_{i}\right)}^{2}}}\right)}^{2}$	
Mean absolute error (MAE):	$\mathrm{MAE}=\frac{\sum_{i=1}^{n}\left|y_{i}-{\tilde{y}}_{i}\right|}{n}$
Pearson correlation coefficient:	
$r_{\mathrm{xy}}=\frac{n\sum_{i=1}^{n}x_{i}y_{i}-\sum_{i=1}^{n}x_{i}\sum_{i=1}^{n}y_{i}}{\sqrt{n\sum_{i=1}^{n}x_{i}^{2}-{\left(\sum_{i=1}^{n}x_{i}\right)}^{2}}\sqrt{n\sum_{i=1}^{n}y_{i}^{2}-{\left(\sum_{i=1}^{n}y_{i}\right)}^{2}}}$	
